# A Rare Cause of Vitamin B12 Deficiency in the Pediatric Age

**DOI:** 10.7759/cureus.61123

**Published:** 2024-05-26

**Authors:** Daniela Couto, Joana Pereira Mendes, Nuno Lourenço

**Affiliations:** 1 Department of Pediatrics, Unidade Local de Saúde da Cova da Beira, Covilhã, PRT

**Keywords:** oral cyanocobalamin, post-traumatic stress, alcohol drinking, vitamin b12 deficiency, adolescent

## Abstract

Vitamin B12 plays an important role in DNA synthesis, hematopoiesis, and neuronal mechanisms, and its deficiency can be associated with insufficient intake or poor absorption and autoimmune and genetic diseases. Although rare, excessive, and chronic alcohol consumption may also justify a deficiency in this vitamin. We present a case of a 17-year-old Iraqi adolescent refugee in Portugal with a deficiency of vitamin B12 due to excessive and chronic alcohol consumption. Although intramuscular (IM) supplementation with cyanocobalamin is the most used, it was decided to initiate oral supplementation. After four months, vitamin B12 levels were replenished. With this clinical case, we aim to raise awareness of the cause of cyanocobalamin deficiency, which although rare in pediatric age should be considered in adolescents, given the possibility of risky behaviors in this age group. Additionally, given the therapeutic options, we have found that oral administration of vitamin B12 has been effective, as reported in the literature.

## Introduction

Vitamin B12 plays an important role in DNA synthesis, hematopoiesis, and neuronal mechanisms [1]. Its deficiency can be associated with insufficient intake or poor absorption, excessive and chronic alcohol consumption, and autoimmune and genetic diseases [2,3]. Treatment consists of supplementation with oral, intramuscular (IM), or nasal cyanocobalamin. Despite the IM route being frequently used, literature data show that oral supplementation is equally effective and is associated with lower costs [4,5].

## Case presentation

A 17-year-old adolescent, Iraqi, institutionalized, and a refugee in Portugal for two years, is being followed up in a Pediatric Psychiatry consultation for posttraumatic stress disorder with associated behavior alteration including episodes of heteroaggressiveness and persecutory speech. He is being treated with olanzapine and alprazolam and has a history of therapeutic noncompliance and multiple missed hospital consultations. An evaluation was requested in the General Pediatrics consultation due to macrocytosis, thrombocytopenia, and vitamin B12 deficiency found in a routine analytical study (Table 1).

**Table 1:** Initial analytical assessment and after four months of vitamin B12 supplementation

	Initial assessment	Re-evaluation after 4 months	Reference values
Leukocytes	4.2 x 10^3^/uL	4.7 x 10^3^/uL	4.0-10.0
Neutrophils	1.6 x 10^3^/uL	2.1 x 10^3^/uL	1.5-8.0
Lymphocytes	2.0 x 10^3^/uL	2.1 x 10^3^/uL	0.8-4.0
Monocytes	0.4 x 10^3^/uL	0.5 x 10^3^/uL	0.0-1.2
Eosinophils	0.1 x 10^3^/uL	0.0 x 10^3^/uL	0.0-0.3
Basophils	0.0 x 10^3^/uL	0.0 x 10^3^/uL	0.0-0.3
Red blood cells	4.49 x 10^6^/uL	4.94 x 10^6^/uL	4.31-6.40
Hemoglobin	15.0 g/dL	16.3 g/dL	13.6-18.0
Mean corpuscular volume (MCV)	97.8 fL	98.9 fL	80.0-97.0
Mean corpuscular hemoglobin (MCH)	33.4 pg	32.9 pg	26.0-34.0
Red cell distribution width (RDW)	12.7 %	12.9 %	11.5-15
Platelets	135 x 10^3^/uL	184 x 10^3^/uL	140-440
Ferritin	62 ng/mL	70.1 ng/mL	30.0-400.0
Folic acid	5.8 ng/mL	5.2 ng/mL	>3.9
Vitamin B12	90.3 pg/mL	329.9 pg/mL	197.0-771.0
Aspartate aminotransferase (AST)	16 U/L	15 U/L	0-38
Alanine aminotransferase (ALT)	10 U/L	9 U/L	0-41
Lactate dehydrogenase (LDH)	155 U/L		125-220
Gamma-glutamyl transferase (GGT)	8 U/L	13 U/L	7-66
Alkaline phosphatase	69 U/L		40-129
Total proteins	7.4 g/dL		6.4-8.3
Albumin	4.7 g/dL		3.2-4.5
Thyroid-stimulating hormone (TSH)	1.85 uUI/mL		0.51-4.30
Free thyroxine (T4)	1.07 ng/dL		0.98-1.63
Anti-parietal cell antibodies		Doubtful	
Anti-intrinsic factor antibodies		Negative	
Anti-transglutaminase IgA antibodies		3.6 U/mL	0.8-10.0
Anti-transglutaminase IgG antibodies		0 U/mL	0.0-10.0

During the first appointment, the adolescent denied gastrointestinal alterations, weight loss, paraesthesia, tremors, or focal neurological deficits. He reported maintaining a varied diet, not vegan/nonvegetarian, only restricting pork consumption. He had no surgical history. He admitted to starting tobacco and alcohol consumption at the age of seven, sometimes drinking a bottle of whiskey per day. Currently, he drinks a few shots of whiskey per week and two beers per day. He smokes about five cigarillos per day. He denied using other drugs or exposure to lithium. Upon objective examination, thinness and muscular atrophy were observed. Mucous membranes were colored. No hyper- or hypopigmented skin patches were observed. No palpable lymphadenopathy. No hepatosplenomegaly. A brief neurological examination, including osteotendinous reflexes, was normal.

A new analytical study was conducted to exclude celiac disease and pernicious anemia. Given the history of multiple missed hospital consultations, it was decided to start oral supplementation with vitamin B12 (1 mg, once a day), and the need to stop alcohol consumption was explained.

The adolescent was re-evaluated four months later, with vitamin B12 levels replenished, no changes in the blood count, no other vitamin deficiencies, and a negative celiac disease screening, negative anti-intrinsic factor antibody, and questionable anti-parietal cell antibody (Table 1). He had no new symptoms, and he maintained the earlier neuropsychiatric manifestations. In addition, he admitted to continuing alcohol consumption, about 140 g of ethanol per week. It was decided to continue supplementation for a period of three months with subsequent analytical re-evaluation. The adolescent missed subsequent appointments, refusing to continue follow-up.

## Discussion

Vitamin B12 is present in most animal-derived products, and although its deficiency is more commonly observed in adults, it can also occur in pediatric age [1]. It can result from inadequate intake or diminished absorption, autoimmune diseases, genetic disorders, and some medications. It is important to note that the drugs the teenager was taking, despite causing sedation, xerostomia, dizziness, and changes in appetite, do not cause a vitamin B12 deficiency [1,2]. In addition, smoking is not a direct cause of vitamin B12 deficiency, but eating habits with less balanced diets can contribute to an increased risk of nutrient deficiency [6].

Although rare, excessive and chronic alcohol consumption may also justify a deficiency in this vitamin. Alcohol interferes with erythropoiesis and the digestion, storage, utilization, and excretion of nutrients [7]. Literature shows that chronic alcohol consumption decreases the absorption of vitamins either by direct damage to stomach and intestinal cells or by inhibiting pancreatic enzymes necessary for the absorption of these elements [8,9]. Moreover, the vitamin deficiency present in these individuals may also be justified by inadequate nutritional intake, as they tend to neglect a balanced and varied diet.

Although this diagnostic hypothesis is the most likely in this clinical case, a study was conducted to exclude pernicious anemia. Indeed, pernicious anemia is one of the main causes of vitamin B12 deficiency. It is an autoimmune disease, where the production of antibodies against intrinsic factors and/or parietal cells prevents the absorption of this vitamin [10]. Although anti-parietal cell antibody was questionable and not specific to pernicious anemia in clinical practice [10], it was decided to conduct a new analytical re-evaluation, but the adolescent did not attend and refused to continue follow-up, thus hindering further evaluation and monitoring.

Clinical manifestations can be relatively nonspecific (Table 2), and it is important to note that although the adolescent was asymptomatic at the first consultations, the deficiency of this vitamin may also explain his neuropsychiatric alterations.

**Table 2:** Clinical manifestations of vitamin B12 deficiency

Type	Clinical manifestations
Hematological	Anemia (macrocytic, megaloblastic), leukopenia, thrombocytopenia, pancytopenia, thrombocytosis
Neuropsychiatric	Cognitive impairment, dementia, depression, gait abnormalities, irritability, olfactory impairment, peripheral neuropathy
Gastrointestinal	Glossitis
Cutaneous	Hyperpigmentation, jaundice, vitiligo

In case of adolescents and adults, the preferred treatment consists of IM supplementation. Additionally, this route is more commonly used in cases of severe anemia, neurological alterations, or compromised absorption, such as in pernicious anemia [4]. However, literature data reveal that the oral route is not inferior to IM administration, has no more adverse effects, and is preferred by patients [11-13]. It is important to mention that some of these studies have limitations, including the use of different administration schemes and doses, short follow-up times, and small samples, with most subjects being above 18 years old, which may explain the limited use of this route in clinical practice [5,9]. A review conducted by Cochrane in 2018, acknowledging the limitations, shows that oral supplementation is equally effective, reducing the need for patient visits to hospitals and associated costs [5].

## Conclusions

Vitamin B12 deficiency can be associated with multiple causes. Given the future consequences such as hematological alterations or neurological impairment, it is essential to make appropriate diagnosis and treatment. With this clinical case, we aim to raise awareness of a cause of cyanocobalamin deficiency, which although rare in pediatric age, should be considered in adolescents, given the possibility of risky behaviors in this age group. Lastly, it is worth emphasizing that given the therapeutic options, we have found that oral administration of vitamin B12 has been effective, as reported in the literature.

## References

[1] Kliegman R, St Geme J (2020). Nelson Textbook of Pediatrics. 21st Edition.

[2] Means Means, R. R., Fairfield Fairfield, K K (2023). Causes and pathophysiology of vitamin B12 and folate deficiencies. UptoDate.

[3] Means R, Fairfield K (2023). Clinical manifestations and diagnosis of vitamin B12 and folate deficiency. Clinical manifestations and diagnosis of vitamina.

[4] Means R, Fairfield K (2023). Treatment of vitamin B12 and folate deficiencies. UpToDate.

[5] Wang H, Li L, Qin LL, Song Y, Vidal-Alaball J, Liu TH (2018). Oral vitamin B(12) versus intramuscular vitamin B(12) for vitamin B(12) deficiency. Cochrane Database Syst Rev.

[6] Deepa Singh (2016). Effect of cigarette smoking on serum homocysteine and vitamin B12 level in male population of Udaipur. Biochem Anal Biochem.

[7] Fragasso A, Mannarella C, Ciancio A, Sacco A (2010). Functional vitamin B12 deficiency in alcoholics: an intriguing finding in a retrospective study of megaloblastic anemic patients. Eur J Intern Med.

[8] (1993). National Institute on Alcohol Abuse and Alcoholism. Alcoholism No. 22 PH 346 October.

[9] Feinman Feinman, l l (1989). Absorption and utilization of nutrients in alcoholism. Alcohol Health Res World.

[10] Mohamed M, Thio J, Thomas RS, Phillips J (2020). Pernicious anaemia. BMJ.

[11] Sanz-Cuesta T, Escortell-Mayor E, Cura-Gonzalez I (2020). Oral versus intramuscular administration of vitamin B12 for vitamin B12 deficiency in primary care: a pragmatic, randomised, non-inferiority clinical trial (OB12). BMJ Open.

[12] Andrès E, Dali-Youcef N, Vogel T, Serraj K, Zimmer J (2009). Oral cobalamin (vitamin B(12)) treatment. An update. Int J Lab Hematol.

[13] Bolaman Z, Kadikoylu G, Yukselen V, Yavasoglu I, Barutca S, Senturk T (2003). Oral versus intramuscular cobalamin treatment in megaloblastic anemia: a single-center, prospective, randomized, open-label study. Clin Ther.

